# The design of RIP belts impacts the reliability and quality of the measured respiratory signals

**DOI:** 10.1007/s11325-020-02268-x

**Published:** 2021-01-07

**Authors:** Kristofer Montazeri, Sigurdur Aegir Jonsson, Jon Skirnir Agustsson, Marta Serwatko, Thorarinn Gislason, Erna S. Arnardottir

**Affiliations:** 1 Nox Research, Nox Medical, 105 Reykjavik, Iceland; 2 Department of Engineering, Reykjavik University, Reykjavik, Iceland; 3 Sleep Department, Landspitali—The National University Hospital of Iceland, Reykjavik, Iceland; 4 Faculty of Medicine, University of Iceland, Reykjavik, Iceland; 5 Internal Medicine Services, Landspitali—The National University Hospital of Iceland, Reykjavik, Iceland; 6 Department of Computer Science, Reykjavik University, Reykjavik, Iceland

**Keywords:** Respiratory inductance plethysmography (RIP) belts, Respiratory airflow, Obstructive sleep apnea (OSA), Signal quality, Signal reliability

## Abstract

**Purpose:**

Evaluate the effect of respiratory inductance plethysmography (RIP) belt design on the reliability and quality of respiratory signals. A comparison of cannula flow to disposable cut-to-fit, semi-disposable folding and disposable RIP belts was performed in clinical home sleep apnea testing (HSAT) studies.

**Methods:**

This was a retrospective study using clinical HSAT studies. The signal reliability of cannula, thorax, and abdomen RIP belts was determined by automatically identifying periods during which the signals did not represent respiratory airflow and breathing movements. Results were verified by manual scoring. RIP flow quality was determined by examining the correlation between the RIP flow and cannula flow when both signals were considered reliable.

**Results:**

Of 767 clinical HSAT studies, mean signal reliability of the cut-to-fit, semi-disposable, and disposable thorax RIP belts was 83.0 ± 26.2%, 76.1 ± 24.4%, and 98.5 ± 9.3%, respectively. The signal reliability of the cannula was 92.5 ± 16.1%, 87.0 ± 23.3%, and 85.5 ± 24.5%, respectively. The automatic assessment of signal reliability for the RIP belts and cannula flow had a sensitivity of 50% and a specificity of 99% compared with manual assessment. The mean correlation of cannula flow to RIP flow from the cut-to-fit, semi-disposable, and disposable RIP belts was 0.79 ± 0.24, 0.52 ± 0.20, and 0.86 ± 0.18, respectively.

**Conclusion:**

The design of RIP belts affects the reliability and quality of respiratory signals. The disposable RIP belts that had integrated contacts and did not fold on top of themselves performed the best. The cut-to-fit RIP belts were most likely to be unreliable, and the semi-disposable folding belts produced the lowest-quality RIP flow signals compared to the cannula flow signal.

**Supplementary Information:**

The online version contains supplementary material available at 10.1007/s11325-020-02268-x.

## Introduction

Sleep studies screening for obstructive sleep apnea (OSA) and other sleep-related breathing disorders generally measure both respiratory flow and respiratory movements [1]. Respiratory inductance plethysmography (RIP) belts are typically used to determine the presence of respiratory effort [1]. Furthermore, dual thoracoabdominal RIP belts are the recommended alternative sensors for the measurement of respiratory events when the primary sensor is not working and provide important information to distinguish between obstructive, mixed, and central apneas as well as obstructive and central hypopneas [1]. RIP measures relative changes in the thoracic and abdomen volume [2], and a signal proportional to flow (RIP flow) can be derived from the RIP signals. The RIP flow can be used either uncalibrated or calibrated [1]; well-calibrated RIP signals will result in a RIP flow signal that better represents the respiratory airflow than uncalibrated signals [3–6].

RIP belt design may potentially affect belt reliability and the quality of the derived RIP flow. The design features of interest include the following: the mechanism by which the belt is fixed to the measurement device, which can influence the contact reliability with the measurement device; whether the RIP belt length is adjusted by folding the belt on top of itself, which can lead to electromagnetic interference [7] and cause artifacts; and whether the belts are washed and reused, which may cause degradation of the material and mechanical properties. The authors did not find any literature describing how design differences might impact the resulting RIP signal.

The aim of this study was to evaluate how different designs of widely used RIP belts are related to their signal reliability and quality. Three different RIP belt types were used: disposable cut-to-fit RIP belts, semi-disposable folding RIP belts, and disposable snap-on RIP belts. The a priori hypotheses were the following: (1) The disposable snap-on belts are more reliable than the disposable cut-to-fit belts, as the snap-on belts have superior contact quality; and (2) The disposable belts have higher signal quality than the semi-disposable belts, as the semi-disposable belts are washed and reused and are adjusted by folding the belt on top of itself.

## Methods

### Patients

This was a retrospective study using 767 clinical home sleep apnea testing (HSAT) studies from adults, ≥ 18 years, performed as part of standard clinical routine at Landspitali, the National University Hospital of Iceland, from January 2009 to May 2017. Consent for this study was granted by the National Bioethics Committee (12-058) and the Data Protection Authority of Iceland.

### Sleep study setup

Patients, referred to a sleep study due to a suspicion of OSA, received a type 3 device for HSAT at an outpatient ward of the hospital. Embletta (Natus, Pleasanton, California, USA) and Nox T3 (Nox Medical, Reykjavik, Iceland) devices were used to record nasal cannula (pressure transducer), thorax, and abdomen RIP and other signals. A sleep technologist configured the device and recorded the relevant patient information, including age, gender, height, and weight. The recording was manually reviewed, the analysis period was marked, and the studies were scored as part of routine clinical work. See the patient hook-up instructions in the Supplementary information.

The disposable cut-to-fit RIP belts were cut to length by the sleep technologist (XactTrace belts, Natus, Pleasanton, California, USA). The belt size of the semi-disposable folding RIP belts and the disposable snap-on RIP belts (Nox Medical, Reykjavik, Iceland) was adjusted as needed by the patient at home.

### Data summary

#### Inclusion criteria:

Clinical HSAT studies at the outpatient ward of Landspitali—The National University Hospital of Iceland.

The original database comprised 767 measurements in three datasets (Table 1).

1. *Disposable cut-to-fit RIP belts:* Embletta studies (Natus, Pleasanton, California, USA) from January 2009 to July 2009.
2. *Semi-disposable folding RIP belts*: Nox T3 studies (Nox Medical, Reykjavik, Iceland) from December 2010 to February 2012.
3. *Disposable snap-on RIP belts*: Nox T3 studies (Nox Medical, Reykjavik, Iceland) from January 2017 to May 2017.

Exclusion criteria: duplicates were removed; cannula, RIP, or oximeter signal files were missing from study; missing patient information; analysis period ≤ 4 h; patient age < 18 years.

**Table 1:** A summary of the number of measurements in each dataset delivered from the hospital

	Disposable cut-to-fit RIP belts	Semi-disposable folding RIP belts	Disposable snap-on RIP belts
Initial count	254	225	288
Measurement duplicated	− 9	− 13	− 1
Missing signal	− 17	− 24	− 2
No patient information	− 13	− 30	− 22
Analysis period < 4 h	− 9	− 9	− 7
Final count	206	149	256

The initial values included all measurements conducted at the hospital in the given time period. The final numbers indicate the number of measurements used in the analysis after excluding the relevant recordings

Abbreviation: *RIP*, respiratory inductance plethysmography

### Signal reliability

An automatic algorithm was generated to determine the reliability of the cannula flow and the thorax and abdomen RIP signals. Unreliable epochs were labeled on the respective channel. A more detailed description of the algorithm and scoring performed is provided in the Supplementary information.

The performance of the algorithm was verified by an experienced sleep technologist, who manually scored a randomly chosen subset of nine sleep studies from each of the three datasets. The sleep technologist (MS) reviewed the recordings in 5-min time frames and was blind to the automatic algorithm scoring. The sleep technologist assessed whether the cannula flow, thorax belt, or abdomen belt independently measured respiratory airflow reliably for clinical scoring of OSA; periods ≥ 10 s were scored as unreliable on the respective signals. All major body movements were excluded from the manual analysis.

### Signal quality

Periods where the cannula flow was scored as unreliable in the signal reliability analysis were excluded from the signal quality analysis. The RIP signals are a qualitative measure of volume. By computing a weighted sum of their derivatives, a signal proportional to flow can be derived [1]. The quality of the RIP flow signals was measured by calculating its correlation to the cannula flow signal. The cannula flow signal was resampled to have the same sampling frequency as the RIP flow signal to allow the correlation between the signal samples to be calculated. The comparison of RIP flow and cannula flow was chosen since high-quality RIP signals should result in accurate measurements of qualitative volume changes which can be used to derive flow. The nasal cannula is in an independent measurement of flow, and it is present in all the sleep studies used in the analysis. To mitigate changes in the signal amplitudes due to subject position changes or sensor displacements, 10-s epochs were investigated. The relative contribution of the thoracic and abdomen RIP signals to the RIP flow signal can impact the signal shape, and calibration techniques can be used to mitigate this [8]. The calibration factors are known to change when a patient changes position or the RIP belts move during sleep [8]. To mitigate this, we opted to tune a scaling factor *x* to maximize the correlation between the calibrated RIP flow signal and the cannula flow signal in each 10-s epoch. This allowed us to determine the maximum theoretical correlation between the signals without being reliant on a specific RIP calibration method. The scaling factor *x* could take values between 0 and 1 for the relative contribution of the time derivative of the thorax RIP (RIP’_th_) and abdomen RIP (RIP’_ab_) belt signals in each epoch.

$$ \mathrm{Calibrated}\ \mathrm{RIP}\ \mathrm{flow}=\left(1-x\right)\ast \mathrm{RIP}{'}_{\mathrm{th}}+x\ast \mathrm{RIP}{'}_{\mathrm{ab}}. $$

A scaling factor *x* close to either 0 or 1 indicated that either the RIP’_th_ or RIP’_ab_ signals did not correlate with the cannula flow signal, and the respective RIP belt might not have been measuring respiratory movements. The Pearson correlation between the cannula and calibrated RIP flow was calculated and *r* reported for each epoch, with *r* > 0.8 assessed specifically [9].

To account for any dissimilarity between the length of the analysis periods in the different recordings, we compared time-normalized quantities by reporting the duration of low reliability periods as a percentage of measurement duration and signal quality as a percentage of total epochs in the dataset.

The reliability and quality algorithms were implemented in Python 3.6.6 using Anaconda 3, NumPy 1.15.2, SciPy 1.1.0, and Pandas 0.23.4.

## Results

### The study cohort

The referred patients were, on average, middle-aged, overweight, and more likely to be male, as expected for sleep study referrals due to a suspicion of OSA. Out of the 611 patients in the final dataset, 37.5% had an apnea-hypopnea index (AHI) < 5.0, 30.4% had AHI 5.0–14.9, 17.0% had AHI 15.0–29.9, and 15.1% had AHI ≥ 30.0. A small but statistically significant difference between the three RIP belt datasets was observed in body mass index (BMI) and oxygen desaturation index (ODI) values (Table 2).

**Table 2:** Summary of patient data for the three RIP belt datasets

	Disposable cut-to-fit RIP belts (*n* = 206)	Semi-disposable folding RIP belts (*n* = 149)	Disposable snap-on RIP belts (*n* = 256)	*p* value*
Demographic data				
Age (years)	50.0 ± 11.4	47.4 ± 12.4	48.5 ± 13.1	0.15
Female (%)	31.1	26.2	33.6	0.30
BMI (kg/m^2^)	29.8 ± 5.7	31.3 ± 5.6	31.9 ± 6.4	0.001
Sleep study				
Analysis period (h)	6.7 ± 1.0	6.9 ± 1.0	6.9 ± 1.9	0.12
AHI (events/h)	14.1 ± 17.6	14.2 ± 19.3	13.6 ± 16.9	0.53
ODI (events/h)	13.9 ± 17.3	12.3 ± 17.7	12.7 ± 16.4	0.04

Mean values are shown as mean ± SD

Abbreviations: *RIP*, respiratory inductance plethysmography; *AHI*, apnea-hypopnea index; *ODI*, oxygen desaturation index

*p values were calculated using one-way ANOVA, except for gender, where a two-way Chi-square test was used. AHI and ODI values were log transformed before hypothesis testing

### Cannula and RIP belt reliability

**Fig. 1:**
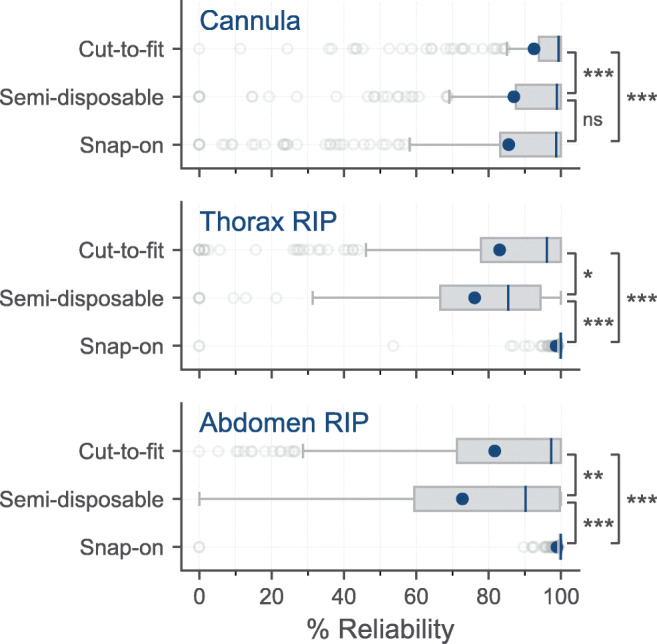
Box plots showing the distribution of the signal reliability values for the three datasets during the analysis period. The median values are indicated with a blue line, the mean values as blue dots, the box edges represent the 25th and 75th percentiles of the data, the whiskers are the range of the data defined as 1.5 times the interquartile length, and the gray circles are considered outliers. The pairwise *p* values, calculated using one-way ANOVA, are shown with *p < 0.05, **p < 0.01, ***p < 0.001, and ns *p* ≥ 0.05. Abbreviation: RIP, respiratory inductance plethysmography

The mean signal reliability of the cannula flow signals was between 85.5 and 92.5% in the datasets, and the distribution had a long tail to the left. Similarly, RIP signal reliability had a distribution with a long tail to the left (Table 3; Fig. 1). This indicates that in most measurements, the cannula and RIP signals were reliable; however, when they were not, the signals tended to be unreliable for a large portion of the measurement. The mean reliability of the disposable snap-on RIP belts was significantly higher than for the disposable cut-to-fit and semi-disposable folding RIP belts (*p* < 0.001). The automatic assessment of signal reliability for the RIP belts and cannula flow had a sensitivity of 50.3% and a specificity of 99.5% compared with manual assessment. See examples of the automatic and manual scoring in Fig. S3.

**Table 3:** Summary data for the three RIP belt datasets

	Disposable cut-to-fit RIP belts (*n* = 206)	Semi-disposable folding RIP belts (*n* = 149)	Disposable snap-on RIP belts (*n* = 256)	*p* value overall comparison
Cannula and RIP belt signal reliability				
Median cannula (%)	99.3 (93.9–100.0)	98.8 (87.6–100.0)	98.7 (83.2–100.0)	
Mean cannula (%)	92.5 ± 16.1	87.0 ± 23.3	85.5 ± 24.5	0.002
Median thorax (%)	96.1 (77.8–100.0)	85.4 (66.6–94.4)	100.0 (99.7–100.0)	
Mean thorax (%)	83.0 ± 26.2	76.1 ± 24.4	98.5 ± 9.3	< 0.001
Median abdomen (%)	97.3 (71.2–97.3)	90.2 (59.4–99.7)	100.0 (99.7–100.0)	
Mean abdomen (%)	81.7 ± 26.8	72.7 ± 36.2	98.8 ± 8.9	< 0.001
Calibrated RIP flow signal quality*				
Median calibrated RIP flow (*r*)	0.89 (0.73–0.94)	0.54 (0.40–0.67)	0.92 (0.84–0.96)	
Mean calibrated RIP flow (*r*)	0.79 ± 0.24	0.52 ± 0.20	0.86 ± 0.18	< 0.001
% calibrated RIP flow > 0.8 (*r*)	67.6%	6.9%	80.6%	

Mean values are shown as mean ± standard deviation and median values as median ± interquartile range

Abbreviation: *RIP*, respiratory inductance plethysmography

*Only calculated for periods in which no artifacts were found in the cannula signal in the signal reliability test

### RIP flow quality

**Fig. 2:**
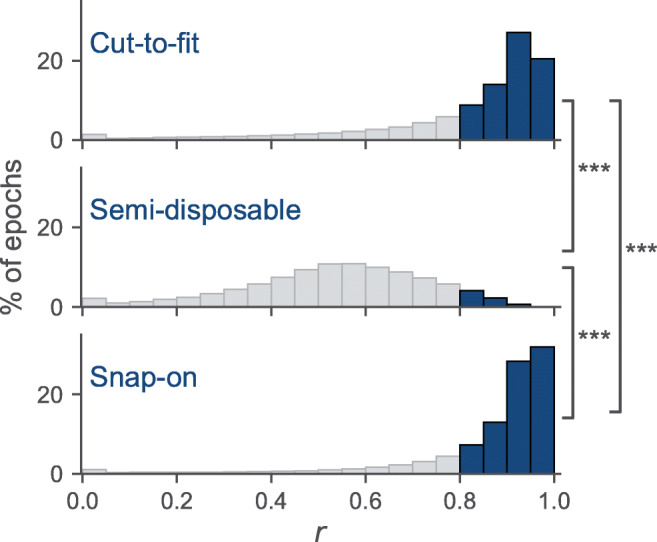
Histograms showing the Pearson correlation between the cannula flow and the calibrated RIP flow in the 10-s data epochs. The bins with *r* values > 0.80 are shown in blue. The Pearson correlation of the RIP flow is highest in the disposable snap-on RIP belt dataset and lowest in the semi-disposable folding RIP belt dataset. The pairwise *p* values, calculated using one-way ANOVA, are shown with ***p < 0.001. Abbreviation: RIP, respiratory inductance plethysmography

The Pearson correlation *r* between cannula flow and calibrated RIP flow was calculated for each epoch in which the cannula and at least one of the thorax or abdomen RIP belts were considered reliable in the analysis above. In the semi-disposable dataset, the distribution leaned more to the left, indicating a low correlation between the cannula flow and the calibrated RIP flow. Only 6.9% of the epochs had a correlation of *r* > 0.8 (Fig. 2; Table 3). Contrarily, the distribution of the *r* values in the disposable cut-to-fit and snap-on datasets leaned to the right, with 67.6% and 80.6% of epochs having *r* > 0.8, respectively (Fig. 2; Table 3).

The correlation between signal quality, age, and BMI showed that there is a slight but statistically significant reduction in signal quality with increased BMI in all three datasets (see Figs. S1 and S2).

**Fig. 3:**
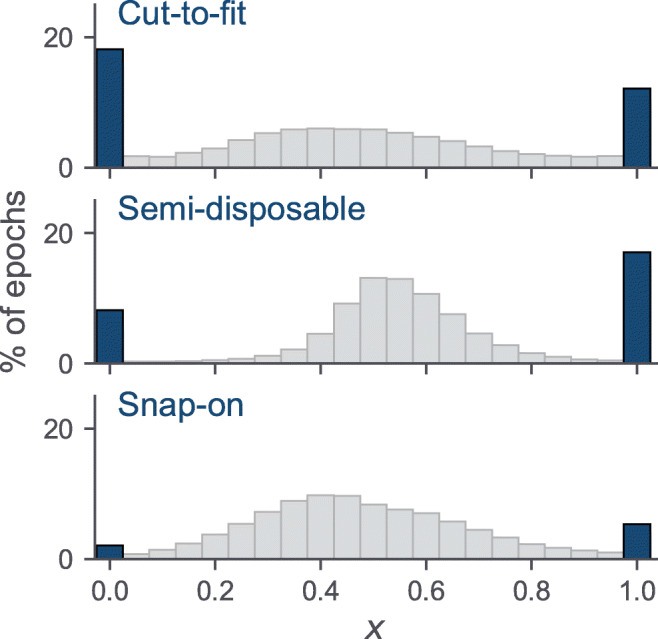
Histograms showing the distribution of the scaling factor *x*, calculating the relevant contribution of the thorax and abdomen belt to the RIP flow in the datasets. The values 0 and 1, shown in blue, indicate no contribution of the thorax and abdomen belt, respectively, to the calculated RIP flow signal. Abbreviation: RIP, respiratory inductance plethysmography

Figure 3 shows histograms of scaling factor *x*. The figure shows that the distribution of the scaling factors had a mean value near 0.5 in the datasets. A scaling factor of either 0 or 1 indicates that the addition of either the signal from the thoracic RIP belt or the abdomen RIP belt decreased the strength of the correlation of the RIP flow signal to the cannula flow signal. When at least one RIP signal was determined to be reliable, a scaling factor of either 0 or 1 was found in 30.3%, 25.2%, and 7.4% of epochs in the disposable cut-to-fit, semi-disposable, and disposable snap-on datasets, respectively. For epochs in which both RIP signals were determined to be reliable, the portion of epochs with a scaling factor of either 1 or 0 was 10.1%, 9.7%, and 7.3%, respectively.

## Discussion

This study shows that the design of the RIP belt can influence the quality of the measured RIP signals in sleep studies. The reliability of the sensors was investigated by determining how frequently the sensors failed to measure respiratory airflow. During periods in which both the cannula and the RIP belts were determined to be reliably measuring respiratory airflow, the quality of the RIP flow signal was determined by calculating the correlation between the cannula and RIP flow signals.

With disposable snap-on RIP belts, the signal reliability was found to be 98.5–98.8% on average, surpassing the reliability of the cannula, which was on average 85.5–92.5% in the different datasets. The most important features for RIP belt reliability are that the conducting wire in the belt does not break and that the connection between the RIP belt and the measuring device is secure. Using the same belts repeatedly can impact the reliability of the RIP belt and cause the wire to break. This can explain the differences in RIP belt reliability found in this study with the best results for disposable snap-on belts but lower reliability for both disposable cut-to-fit belts (likely due to connection issues) and semi-disposable folding belts (likely due to repeated use).

We consider that the main factor which influences the RIP signal quality is when the length of the RIP belts is adjusted by folding the belt onto itself. When a RIP belt is folded onto itself, the measured signal is corrupted by electromagnetic interference [7]. The impact of electromagnetic interference depends on how the ripples in the RIP belt wires line up against each other and how they move relative to each other during breathing. The semi-disposable folding RIP belt was designed in this way, and the flow signal measured correlated poorly with the cannula flow.

The strengths of this study include that the data originated from the same clinical source, the study was performed with comparable protocols, reducing bias due to the use of different protocols. The data were collected during routine clinical practice, reflecting normal use of the sensors with a large dataset. The calculation of the calibrated RIP flow signal depended only on the similarity of the RIP flow signal to the cannula flow signal, independent of device or sensor manufacturer. The limitations of the study include possible changes in the years during which data were collected (e.g., in the patient population and patient sleep study instructions), which the study could not control for and may effect both the cannula and RIP signal assessments. The comparison of flow derived from the RIP belts to cannula flow has limitations in the presence of mouth breathing; this effect should be equal in all datasets. A comparison between oronasal pneumotachography and different RIP belt designs, performed at the same time point, would be useful to confirm the results of this study. One limitation is the determination of signal reliability. No standard definition of signal reliability exists, and determining one is challenging. To gauge the performance of the automatic assessment of signal reliability, a sleep technologist reviewed the signals and labeled periods during which it was impossible to detect respiratory events in a small subset of the sleep studies. The automatic algorithm estimated signal reliability by looking at signal amplitudes and determining whether signals in the sensors originated from the same source. The automatic assessment of signal reliability for the RIP belts and cannula flow had a sensitivity of 50.3% compared with manual assessment and specificity of 99.5%. This indicates that the automatic algorithm was more tolerant of low signal quality than the technician was; however, when the algorithm detected low quality, it was confirmed by manual assessment (Fig. S3). Using the same algorithm on all three datasets reduces the likelihood of bias. Finally, in this study, we did not assess the effect of RIP belt design on the measured AHI as this was considered out of scope to the current work. The agreement of scoring respiratory events using RIP belts in general and signals from different sensors: oronasal pneumotachograph, oronasal thermal sensors, and nasal cannulas, has been previously reported [8, 10–13]. They show that the agreement between manual scoring of respiratory events using an oronasal thermal sensor or nasal cannula and RIP belts is high on average, Pearson correlation (*r*) of 0.88 [10] and intraclass correlation (ICC) of 0.97 [11]. However, the classification of respiratory events into apneas and hypopneas is not as good. One study found an intraclass correlation for the apnea index of 0.66 and for the hypopnea index of 0.79 [11]. The effect of RIP belt quality on the scoring agreement remains to be answered in future studies.

The study shows that RIP belts design impacts the reliability and quality of the measured signals.

## Supplementary Information

ESM 1 (DOCX 901 kb)

## Abbreviations

AASM: American Academy of Sleep Medicine
AHI: Apnea-hypopnea index
ANOVA: Analysis of variance
BMI: Body mass index
HSAT: Home sleep apnea testing
ICC: Intra-class correlation
ODI: Oxygen desaturation index
OSA: Obstructive sleep apnea
RIP: Respiratory inductance plethysmography
SD: Standard deviation
