# Supplementary material for: The design of RIP belts impacts the reliability and quality of the measured respiratory signals
Source: Sleep Breath. 2021 Jan 7;25(3):1535–41. doi: 10.1007/s11325-020-02268-x (PMC8376735; doi:10.1007/s11325-020-02268-x)
Supplement: Supplementary file 1 — (DOCX 901 kb) [file 11325_2020_2268_MOESM1_ESM.docx]

Title: Supplementary materials – The design of RIP belts impacts the reliability and quality of the measured respiratory signals.

Authors: Kristofer Montazeri, Bsc^1^; Sigurdur Aegir Jonsson, PhD^1^; Jon Skirnir Agustsson, PhD^1^; Marta Serwatko, Msc^2^; Thorarinn Gislason, PhD, MD^3,4^ and Erna S. Arnardottir, PhD^2,5^.

Affiliations:

1. Nox Research, Nox Medical, Reykjavik, Iceland.
2. Department of Engineering, Reykjavik University, Reykjavik, Iceland
3. Sleep Department, Landspitali – The National University Hospital of Iceland, Reykjavik, Iceland.
4. Faculty of Medicine, University of Iceland, Reykjavik, Iceland.
5. Internal Medicine Services, Landspitali – The National University Hospital of Iceland, Reykjavik, Iceland.

Corresponding author:

Dr. Erna Sif Arnardottir

Department of Engineering

Reykjavik University

102 Reykjavik, Iceland

ernasifa@ru.is

**Methods**

Sleep study setup

The patient hook-up instructions were similar for the data collected with cut-to-fit and semi-disposable respiratory inductance plethysmography (RIP) belts, in-lab instructions by a sleep technologist. However, when the recordings with disposable snap-on RIP belts were performed, the practice of the sleep lab at the hospital had changed. Typically, the patient did not receive in-lab instructions by a sleep technologist but rather set the device up at home with the help of an online video. In these cases, the sleep technologists met with each patient to include the correct size of belts and to ensure that the patients were comfortable with online instructions only. Patients not willing or able to set up the device with the assistance of a video could receive in-lab instructions.

The Embletta studies were originally performed and scored with Somnologica software and Nox T3 studies with the Noxturnal software. All the studies were scored using the latest guidelines from the American Academy of Sleep Medicine (AASM)^1^ at the time of the performed sleep study. All studies were scored hypopneas with ≥30% reduction in flow and ≥4% desaturation. The cannula was used as the primary sensor for apneas and hypopneas and RIP flow as a secondary sensor.

The nasal cannula is the primary sensor for flow in the manual by the AASM^1^ when scoring hypopneas and a recommended sensor when oronasal thermal airflow sensor is not reliable when scoring apneas. The nasal cannula is assumed to be consistent across the devices and timespan of the measurement. Therefore, the nasal cannula was used as the reference flow signal in this study. Only recording periods within a marked analysis period as scored manually by a sleep technologist (from analysis start at the beginning of the night to analysis stop at the end of the night) were used.

Signal reliability

1. *Preprocessing stage:* Included only the study period labeled as the analysis period by the sleep technologist scoring the sleep studies. The flow and RIP belt signals were bandpass filtered between 0.01 Hz – 5.00 Hz and each signal split into 10 second epochs.
2. *Processing stage:* Every 10 second epoch of each signal was classified as reliable or not. The classification algorithm checked the following:

- The power of each signal in the frequency band 0.1 - 2.0 Hz relative to the power of the signal in the frequency band from 0.1 – 5.0 Hz. If the ratio of the powers was less than to 0.5, the signal was assumed to contain only noise in the epoch. The normal respiratory rate of humans lies around 0.3 Hz^2,3^ and a substantial part of the power of the respiratory signals should be around the frequencies of respiration. If the signal contains only white noise the power ratio would be around 0.39 because white noise is defined as noise having equal power at all frequencies (2.0-0.1) / (5.0-0.1) ≈ 0.39.
- The standard deviation of the nasal flow measured by a cannula. If the standard deviation was below 0.015 cmH_2_O, the signal was assumed to contain only noise in the epoch. The threshold value was chosen to be close to the noise floor of the cannula flow signal.
- The maximum coherence between the respiratory signals on the frequency band 0.2 Hz – 2 Hz. The frequency band was chosen because it encloses typical respiratory rates and their first harmonics.^2,3^ Coherence is a measure of how synchronized two signals are.^4^ Signals originating from the same source, respiration in this case, will have a high coherence value at frequencies around the respiratory rate of the patient. If the maximum coherence value between a pair of respiratory signals was above 0.8, the signals were assumed to originate from the same source, i.e. the respiratory movements.

1. Postprocessing stage: Automatic scoring with the default Respiratory Cannula Flow analysis in the Noxturnal software version 5.1 (Nox Medical, Reykjavik, Iceland). The classifications of all epochs of each signal was postprocessed in the following way and in the following order:

- Nasal flow was not classified as being unreliable if there was negative correlation between thorax and abdomen (presumed due to paradoxical respiratory movements).
- If the automatic scoring scored the following events: oxygen desaturation, mixed or central apneas during the epoch that was classified to be of low quality, the epoch was reclassified to be reliable.
- The classifications for each signal were filtered with a median filter^5^ of length 19 10 second epochs (190 seconds). The median filter assigned a value to each epoch as the median value of the 9 epochs preceding the epoch, the current epoch, and the 9 epochs following it. This ensured that periods of 9 or fewer epochs marked unreliable were removed if the surrounding epochs were reliable in the signal, or periods marked as reliable were removed if the signal was in majority scored as being unreliable.
- All periods ≥10 seconds marked as movement were also marked as unreliable in the recording and not included in analysis period of the respective signal.

**Results**

Correlation between parameters

Scatter plots were made to investigate if correlation was present between the signal reliability, and age and BMI. The scatterplots shown below illustrate there is little to no correlation between the parameters.

| (A) | (B) | (C) |
| --- | --- | --- |
| 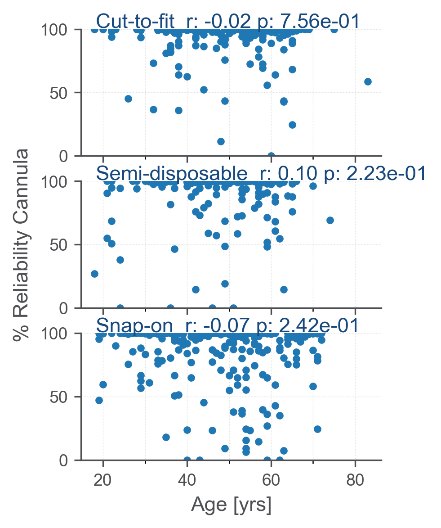 | 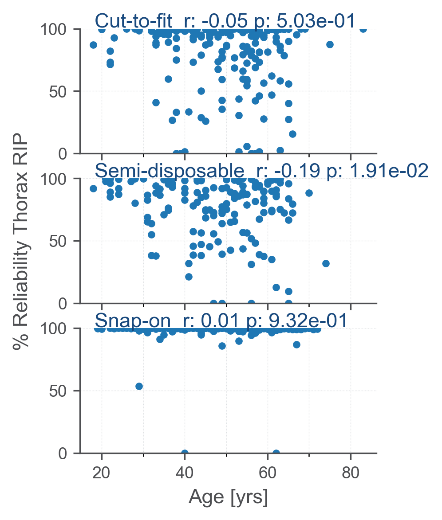 | 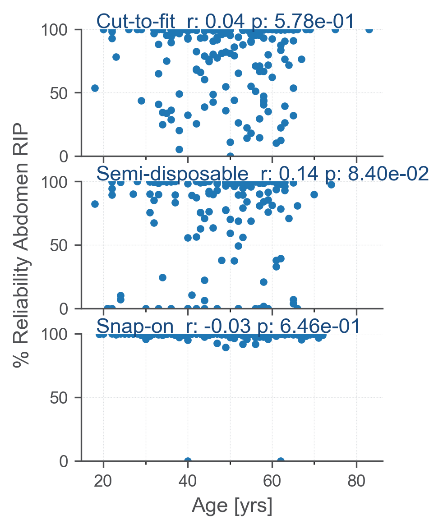 |
| (D) | (E) | (F) |
| 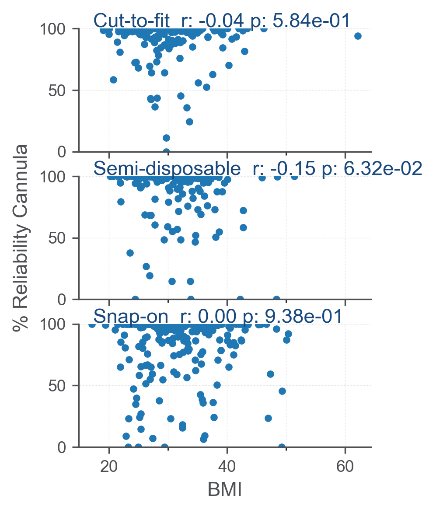 | 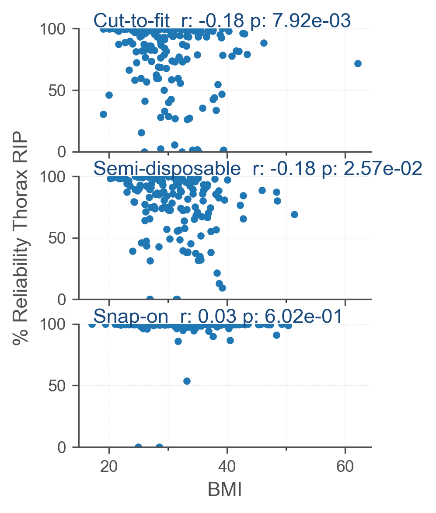 | 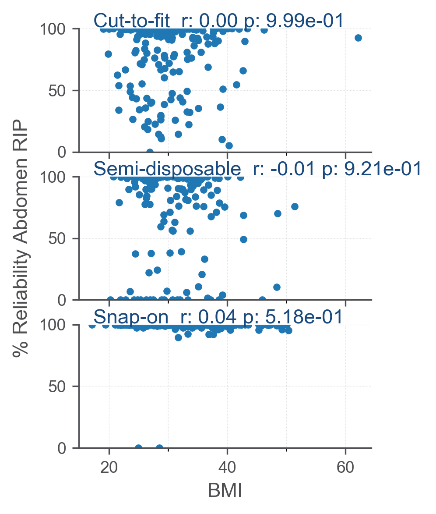 |

Figure S1: Scatter plots of signal reliability and (A)-(C) age, and signal reliability and (D) – (E) BMI.

Scatter plots were made to investigate if correlation was present between the RIP flow signal quality, and age and BMI. The scatterplots shown below illustrate there is small but statistically significant correlation between the *r* and BMI parameters.

| (A) | (B) |
| --- | --- |
| 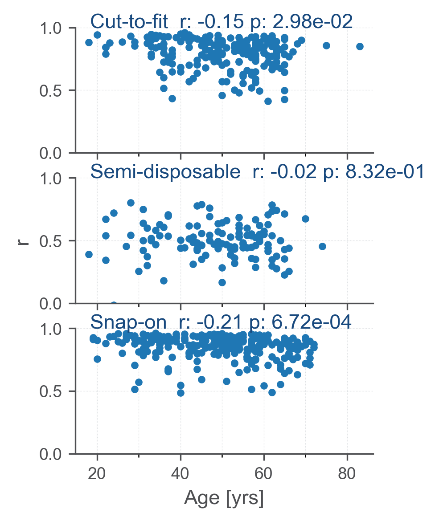 | 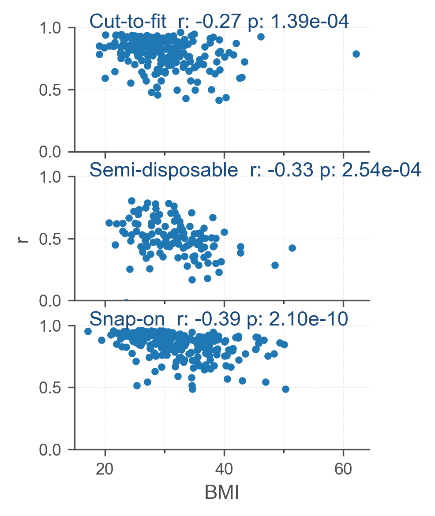 |

Figure S2: Scatter plots of RIP flow quality *r* and (A) age, and (B) BMI.

Manual and automatic scoring of signal quality

(A)


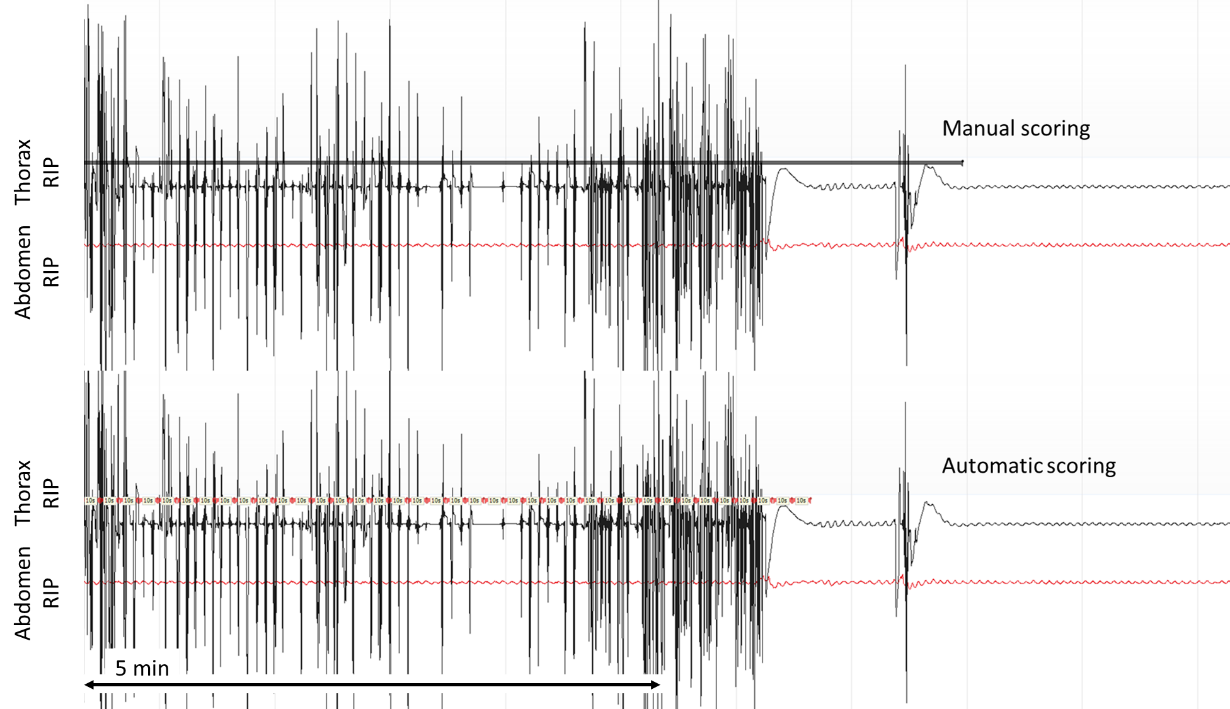


(B)


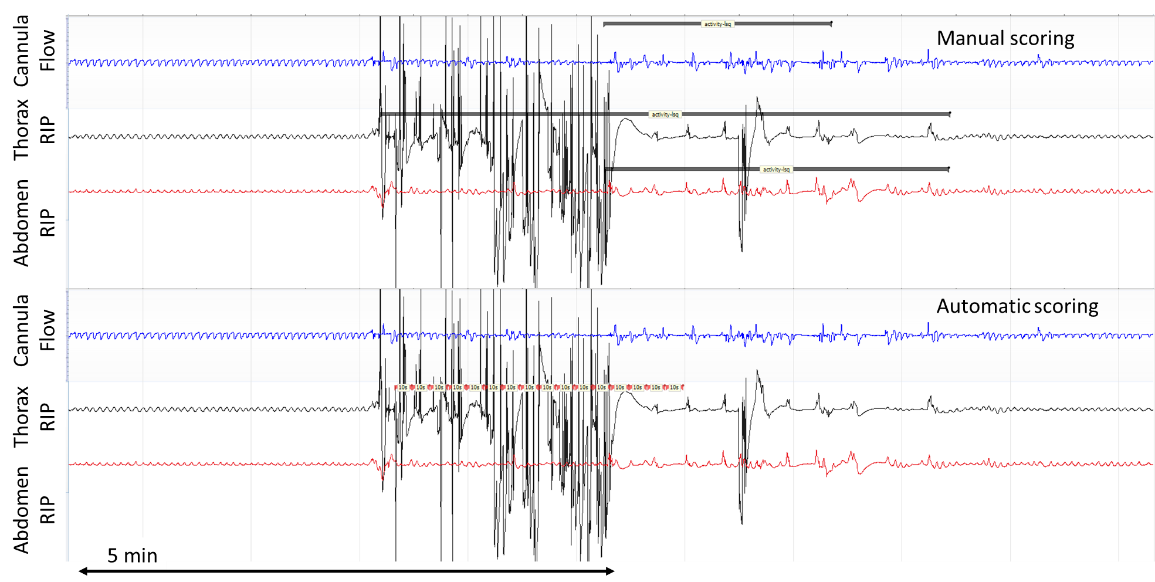


(C)


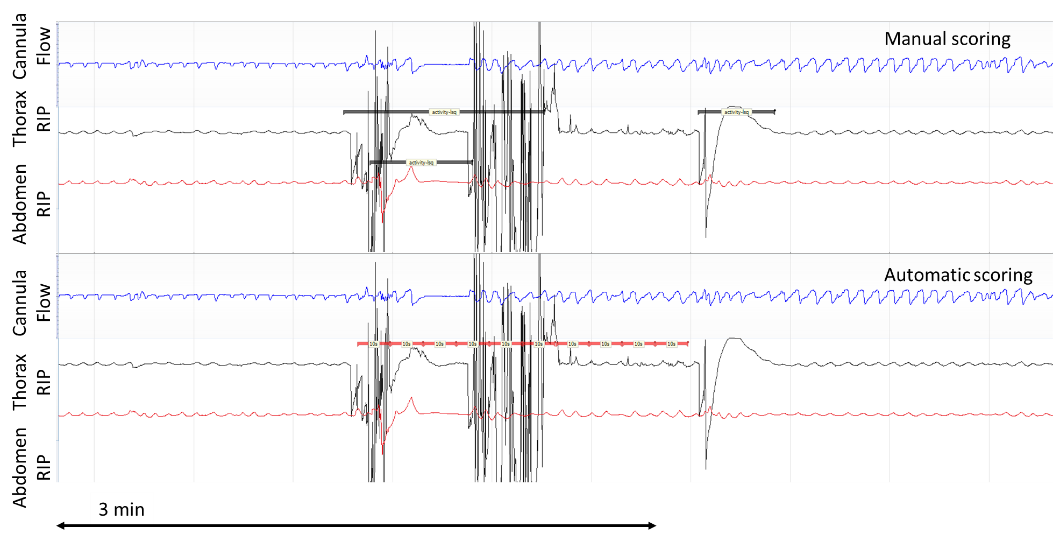


Figure S3: Screenshots from cannula flow, thorax, and abdomen RIP signals showing the manual and automatic scoring of low signal quality and how they could differ in length and occurrence. In the figures, the thoracic RIP signal has instances of unreliable signals where the manual and automatic scoring agrees. In other cases of unreliable signals, in the thoracic and abdomen RIP, there are more manually scored events than automatically scored events.

**References**

1. Berry RB, Albertario CL, Harding SM, et al. *The AASM Manual for the Scoring of Sleep and Associated Events; Rules, Terminology and Technical Specifications, Version 2.5*. American Academy of Sleep Medicine; 2018.

2. Rodríguez-Molinero A, Narvaiza L, Ruiz J, Gálvez-Barrón C. Normal respiratory rate and peripheral blood oxygen saturation in the elderly population. *J Am Geriatr Soc*. 2013;61(12):2238-2240. doi:10.1111/jgs.12580

3. Lindh WQ, Pooler M, Tamparo CD, Dahl BM. *Delmar’s Clinical Medical Assisting*. 5th ed. Cengage; 2014.

4. Bendat JS, Piersol AG. *Random Data: Analysis and Measurement Procedures*. 4th ed. Wiley; 2010.

5. Tukey JW. *Exploratory Data Analysis*. 1 edition. Reading, Mass: Pearson; 1977.
